# Seasonally distinct taxonomic and functional shifts in macroinvertebrate communities following dam removal

**DOI:** 10.7717/peerj.3189

**Published:** 2017-04-06

**Authors:** S. Mažeika P. Sullivan, David W.P. Manning

**Affiliations:** School of Environment and Natural Resources, The Ohio State University, Columbus, OH, United States

**Keywords:** Aquatic invertebrates, Diversity, Lowhead dams, River restoration, Thresholds

## Abstract

Dam removal is an increasingly popular restoration tool, but our understanding of ecological responses to dam removal over time is still in the early stages. We quantified seasonal benthic macroinvertebrate density, taxonomic composition, and functional traits for three years after lowhead dam removal in three reaches of the Olentangy River (Ohio, USA): two upstream of former dam (one restored, one unrestored), and one downstream of former dam. Macroinvertebrate community density, generic richness, and Shannon–Wiener diversity decreased between ∼9 and ∼15 months after dam removal; all three variables consistently increased thereafter. These threshold responses were dependent on reach location: density and richness increased ∼15 months after removal in upstream reaches versus ∼19 months downstream of the former dam. Initial macroinvertebrate density declines were likely related to seasonality or life-history characteristics, but density increased up to 2.27× from year to year in three out of four seasons (late autumn, early spring, summer) across all reaches. Macroinvertebrate community composition was similar among the three reaches, but differed seasonally based on non-metric multidimensional scaling (NMDS) and analysis of similarity (ANOSIM). Seasonal differences among communities tended to decrease after dam removal. We detected community-wide shifts in functional traits such as multivoltinism, depositional habitat use, burrowing, and collector-gatherer feeding mode. We observed that these traits were expressed most strongly with Chironomidae, which was the most abundant family. Our results suggest that seasonal environmental conditions can play a role in the response and recovery of macroinvertebrate communities—often used to monitor ecosystem condition—following dam removal. In particular, macroinvertebrate density and diversity can show recovery after dam removal, especially in seasons when macroinvertebrate density is typically lowest, with concomitant changes to functional trait abundance. Thus, we recommend scientists and managers consider responses to dam removal throughout the year. Further, similar density, generic richness, and functional traits among reaches suggest that channel restoration after dam removal may initially have equivocal effects on invertebrate communities.

## Introduction

The timing of hydrologic disturbances control the physical and chemical template that structure communities in fluvial systems (Stanley, Powers & Lottig, 2010). However, streamflow predictability (Poff & Ward, 1989) can be disrupted by infrastructure such as dams (Hart et al., 2002; Doyle et al., 2005) that inhibit natural flow regimes and sediment transport, restructure aquatic systems from lotic to lentic environments, and limit high-quality habitat availability, dispersal, and gene flow for river biota (Dynesius & Nilsson, 1994; Ligon, Dietrich & Trush, 1995; Roberts, Angermeier & Hallerman, 2013). Considerable attention has been directed towards the ecological effects of large dams (Buckley, 2003; Pess et al., 2008); however, the ecosystem consequences of lowhead, run-of-river dams have received less consideration (Mbaka & Mwaniki, 2015; Gartner, Magilligan & Renshaw, 2015; Magilligan et al., 2016) despite their ubiquity (e.g., ∼50% of dams surveyed in the continental United States are <8 m in height) and impacts on multiple aquatic taxa including fish (Tiemann et al., 2004; Helms et al., 2011; Magilligan et al., 2016), mussels (Tiemann et al., 2007; Gangloff et al., 2011; Smith, Edds & Goeckler, 2015) and insects (Tiemann et al., 2005; Martínez et al., 2013; Mbaka & Mwaniki, 2015).

The short-term release of sediments formerly stored behind dams is a key perturbation associated with lowhead dam removal (Doyle, Stanley & Harbor, 2003; Magilligan et al., 2016), but over the longer-term (i.e., years) there may be reestablishment of seasonally driven disturbance regimes (e.g., frequent scouring flows in late autumn through spring). From this perspective, the effects of dam removal are two-fold, in terms of the initial effects on the physical structure of the river (pulse disturbance; Tullos, Finn & Walter, 2014; Dorobek, Sullivan & Kautza, 2015; Gartner, Magilligan & Renshaw, 2015) and the reestablished seasonal disturbances from high flows over longer timescales (press or ramp disturbance; e.g., Maloney et al., 2008). Despite evidence that dam removal effects can occur at both short and longer time scales, few studies have examined the trajectory of ecological responses over multiple years and across different seasons within the same system (but see Hansen & Hayes, 2012).

Benthic macroinvertebrate communities undergo seasonal shifts (Merritt, Cummins & Berg, 2008) and can respond rapidly to disturbances, including those driven by dam removal (Orr et al., 2008; Renöfält et al., 2013; Tullos, Finn & Walter, 2014). Macroinvertebrate densities and taxonomic richness have been shown to decline after discrete high-flow events (i.e., scouring floods; Gjerløv, Hildrew & Jones, 2003; Suren & Jowett, 2006) and dam removal (Orr et al., 2008; Hansen & Hayes, 2012; Renöfält et al., 2013). Likewise, relative functional trait abundance can be affected by frequent high-flow events whereby taxa that are tolerant to unstable conditions become more established (Wallace, 1990; Townsend, Scarsbrook & Doledec, 1997; Suren & Jowett, 2006). In contrast, dam-removal effects on macroinvertebrate taxonomic structure and functional traits can be idiosyncratic and less predictable. For example, Renöfält et al. (2013) found that although some taxa were unaffected by dam removal or recovered quickly, others showed continuous declines, likely due to site-specific differences in tolerance to sediment deposition and recolonisation. Tullos, Finn & Walter (2014) observed that functional assemblages recovered within one year following lowhead dam removal, and trait modalities reflected both habitat stability (e.g., univoltinism) and disturbance (e.g., clinging habit).

Despite the increasing popularity of dam removal as a river-restoration technique (O’Connor, Duda & Grant, 2015), the interactions among dam-removal perturbations, long-term reestablishment of longitudinal connectivity, and seasonal patterns of macroinvertebrate density and community structure have not been fully considered. Additionally, studies providing direct comparisons of channel restoration vs. no channel restoration following dam removal are lacking (but see Katopodis & Aadland, 2006). Evaluation of macroinvertebrate communities is a well-established and ubiquitous management tool that integrates long-term consequences of perturbations (Wallace, Grubaugh & Whiles, 1996), making their use as indicators of dam removal effects applicable to an array of restoration and river-management scenarios.

Rather than a before-after study, our aim was to investigate the responses of aquatic macroinvertebrates at 10 seasonally distinct intervals over three years following the removal of a lowhead dam on the Olentangy River in Columbus, Ohio. Given limitations in experimental designs common in dam removal studies (see Methods for additional details), we framed our research as exploratory, guided by the following questions: (1) How do macroinvertebrate community density and diversity respond in the initial years following lowhead dam removal?, (2) Are these responses divergent above and below the previous dam, and between restored and unrestored river reaches?; and (3) Are shifts in macroinvertebrate communities seasonally dependent? In addressing these questions, we anticipate that this work will contribute to the growing understanding of the ecological effects of dam removal, as well as to its utility as a river-management tool.

## Materials and Methods

### Study system and experimental design

**Figure 1:**
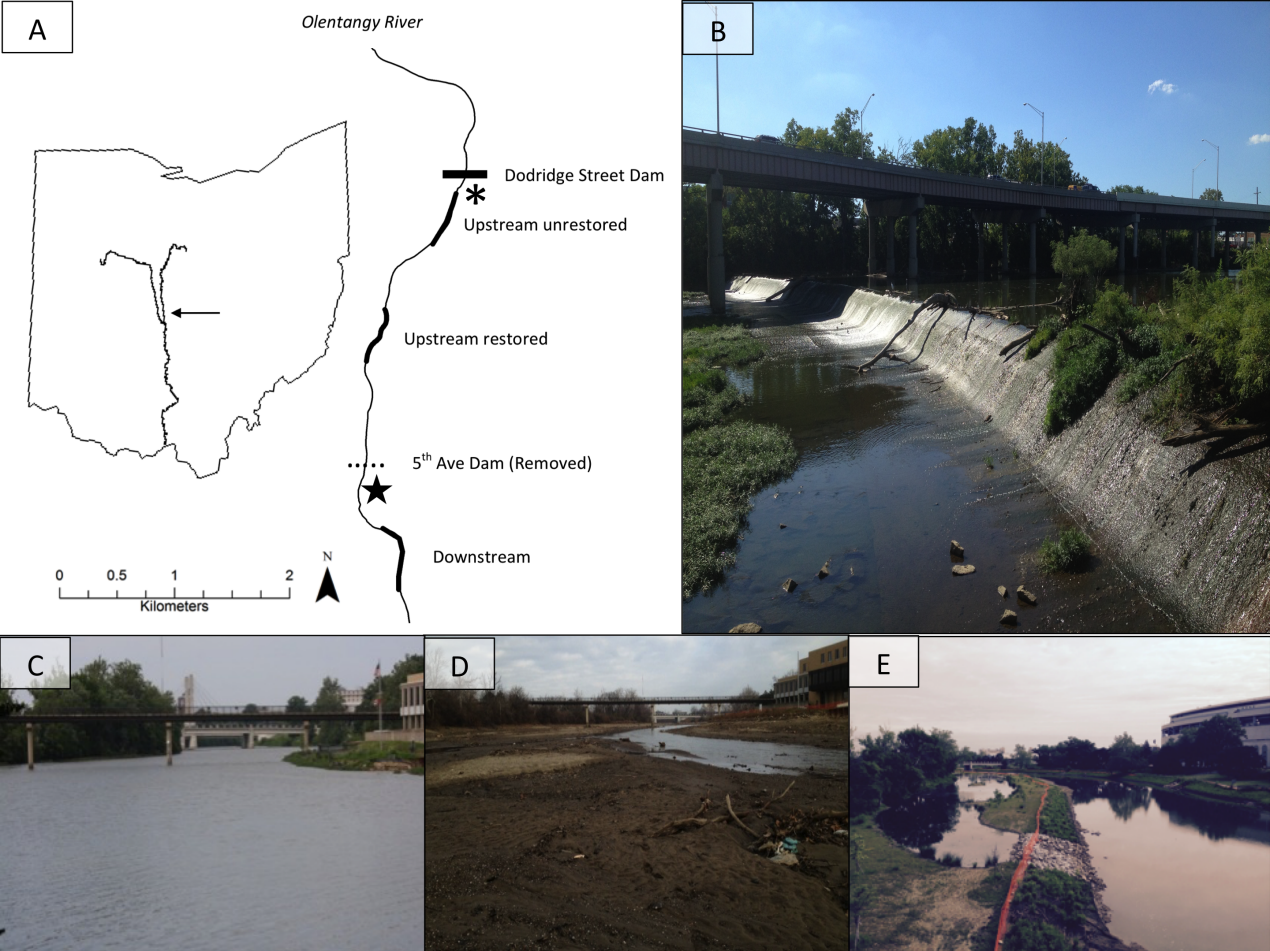
The map (A) shows study site locations on the Olentangy River, and (B) shows the former dam. The remaining photographs depict views upstream of the 5th Avenue Dam overlooking the upstream-restored reach on the Olentangy River, Columbus, Ohio before (C), 2 months (D), and 36 months (E) after dam removal. Thicker lines in (A) denote the study sites: upstream unrestored, upstream restored, and downstream. The horizontal dotted line indicates the location of the former dam, and the horizontal solid line indicates the location of an intact lowhead dam (Dodridge Street Dam), directly downstream of which is the from Vent (2015). Photo credits: SMP Sullivan.

The 5th Avenue Dam (∼2.4-m high, and 143-m wide) was removed in September 2012 to improve water quality and aquatic habitat in the 5th-order Olentangy River, Ohio, USA (39°59′N, 83°01′W; US Army Corps of Engineers, 2004; Fig. 1A). We studied effects of its removal upstream and downstream using three 360-m study reaches: one reach ∼400 m downstream of previous dam location, and two reaches upstream of the previous dam (i.e., the former reservoir) (Fig. 1B). One of the upstream reaches (∼1,300 m from previous dam) was restored after dam removal (hereafter: upstream restored; Figs. 1C–1E), including river-channel engineering to redevelop and reconnect floodplain wetlands (Ohio Environmental Protection Agency, 2011). The second upstream reach (∼2,300 m from previous dam) was allowed to adjust naturally (hereafter: upstream unrestored). Prior to dam removal, the downstream reach was a shallow riffle whereas the two upstream reaches were characterised by deeper, slower moving-to-impounded water, and wide channel widths (e.g., Fig. 1C). We collected samples from three sub-sites located at the upstream, middle, and downstream sections of each study reach for sub-site level replication (see Methods: Benthic Macroinvertebrates).

Common logistical constraints associated with studying dam removal were associated with our study; for example, we were unable to collect comparable benthic macroinvertebrate samples given the depth and benthic conditions in the study reaches prior to dam removal (see Methods: Benthic Macroinvertebrates; Fig. 1C). Nevertheless, our study encompassing three impact reaches with multiple samples in space and time is consistent with dam-removal study designs in the literature (Kibler, Tullos & Kondolf, 2011). Although we had no true control reach, fish-community data from a reach above an intact dam in the same river generally showed no differences before and during the three years following dam removal (Dorobek, Sullivan & Kautza, 2015), supporting dam removal as a major driver of ecological shifts in our study reaches. We also considered benthic-macroinvertebrate community data collected from a companion study in a reach directly below an intact dam in the Olentangy River (Fig. 1A; Vent, 2015). We used these data to represent the reference state of benthic macroinvertebrates in the study system to evaluate the practical significance (Kibler, Tullos & Kondolf, 2011) of dam removal effects on benthic macroinvertebrates relative to observations below an intact dam during the same time as our study. These reference data were collected in June, August, and November 2014, and March and June 2015 using comparable methods to those outlined below (i.e., Surber samplers; see Methods: Benthic macroinvertebrates, Vent, 2015).

### Benthic macroinvertebrates

We established three transects (upstream, middle, downstream) extending laterally across the river channel (i.e., from left to right bank) at each study reach. Benthic macroinvertebrates were collected at random points along each transect using a 0.30-m^2^ Surber sampler (500-µm mesh) for 90 s following Sullivan & Watzin (2008) (*n* = 3 per study reach). Starting in June 2013, we consecutively sampled each reach 10 times over three years in four seasons: late autumn (December 2013 and November 2014), early spring (April 2014 and 2015), late spring (June 2013 and 2014), and summer (August 2013, 2014, 2015) capturing both seasonal high (spring) and low (summer, late autumn) flows (see also Fig. S1). Note that we also opportunistically sampled in July 2014. Samples were preserved in 70% ethanol, and identified to the lowest taxonomic resolution possible by Rhithron Associates, Inc. (Missoula, Montana, USA). The most common taxonomic resolution was genus, including genus-level identification for Chironomidae in 98% of cases. Invertebrates were collected under Ohio Division of Wildlife Wild Animal Permit 15-49.

### Numerical and statistical analysis

We calculated macroinvertebrate generic richness (*R*), Shannon–Wiener Diversity Index (*H*′), and evenness (*E*) for the three transects at each study reach and time interval (except for in Jun–Dec 2013 when subsamples were mistakenly combined), from which we computed a reach-specific mean and standard error. The Shannon–Wiener Diversity Index is indicative of both taxonomic richness and evenness, such that,

(1) \begin{eqnarray*}{H}^{{^{\prime}}}=-\sum _{i=1}^{g}{p}_{i}\ast ln\;{p}_{i}\end{eqnarray*}

where *p*_*i*_ is the proportion of the total sample represented by genus (*g*) *i*. We computed generic evenness as

(2) \begin{eqnarray*}E= \frac{{H}^{{^{\prime}}}}{{H}_{max}} \end{eqnarray*}

where *H*′ is the Shannon-Wiener Diversity Index, and *H*_*max*_ is the natural log of generic richness, such that evenness ranges from 0 to 1, with values approaching 1 indicating greater generic evenness in the community.

We also quantified the functional structure of the macroinvertebrate communities after dam removal (Tullos, Finn & Walter, 2014). We assigned each taxon to eight life-history (e.g., voltinism), morphological (e.g., attachment, armoring), and ecological (e.g., functional feeding groups, rheophily) traits, using genus-level classifications according to Poff et al. (2006) (Tables S1 and S2). We selected traits based on their expected ability to indicate physical or ecological changes associated with dam removal (e.g., predominant attachment traits could indicate altered streamflow velocities).

For macroinvertebrate density, taxonomic richness, diversity, and evenness, we used general linear models (GLMs) with time and reach as predictor variables where both were considered fixed effects. Visual inspection of our data revealed potential non-linear responses to dam removal through time. In these cases, we used breakpoint regression (Muggeo, 2003; Dodds et al., 2010) to estimate possible threshold responses (i.e., point at which there is an abrupt ecological change; Dodds et al., 2010). We compared evidence for linear models vs. piece-wise linear models using Davies tests (Davies, 1987; Davies, 2002) to explicitly evaluate non-zero differences in slope through time. We also tested for differences in mean macroinvertebrate density between or among comparable seasons (e.g., late spring 2013 vs. late spring 2014) categorically using orthogonal contrasts (Dowdy, Wearden & Chilko, 2004). Data were ln-transformed (or ln[*x* + 1] in the case of relative abundance) to meet assumptions of normality and homogeneity of variance when deemed appropriate after visual inspection of residuals versus fitted values.

We used non-metric multidimensional scaling (NMDS; Clarke, 1993) followed by analysis of similarities (ANOSIM) to assess differences in macroinvertebrate communities among the three reaches, and through successive months after dam removal. NMDS incorporated Bray-Curtis dissimilarities between relative densities (no. ind m ^−2^/total no. ind m^−2^) of genera for a given month or reach and was limited to a two-dimensional solution. To evaluate community shifts through time, we used the function *ordiellipse* in R to compute centroids of ellipses around ordination points grouped by month. We then computed the Euclidian distance traveled by each ellipse from year to year, and compared these distances between years within seasons ad hoc. We also compared each seasonally distinct centroid to the final sampling date centroid (summer 2015).

In an effort to account for patterns attributable to the dominance of specific macroinvertebrate families, we conducted NMDS and ANOSIM with and without Chironomidae, the most abundant family observed throughout the study (see below: Results). Chironomidae is a taxonomically and functionally diverse family (>1,000 species in North America; Ferrington, Berg & Coffman, 2008) that occurs in high densities, suggesting that changes to their relative abundance might contribute disproportionately to observed responses. Lastly, given the potential lack of independence among study reaches, we tested for spatial autocorrelation (Moran’s *I*) among response variables, and found no evidence for non-random spatial patterns in benthic macroinvertebrate responses including density, richness, and diversity (*P* > 0.05 in all cases).

All statistical analyses were performed using R statistical software v.3.3.0 (R Core Team, 2016). We used the R package ‘segmented’ to estimate possible breakpoints in the relationship between macroinvertebrate variables and months after dam removal (Muggeo, 2003; Muggeo, 2008). We used the R package ‘vegan’ (Oksanen et al., 2013) to generate and analyse ordination data for macroinvertebrate communities through time, and among our study reaches. In all cases, *P* < 0.05 was considered evidence of statistical significance, and *P* < 0.10 was considered evidence of a trend.

**Figure 2:**
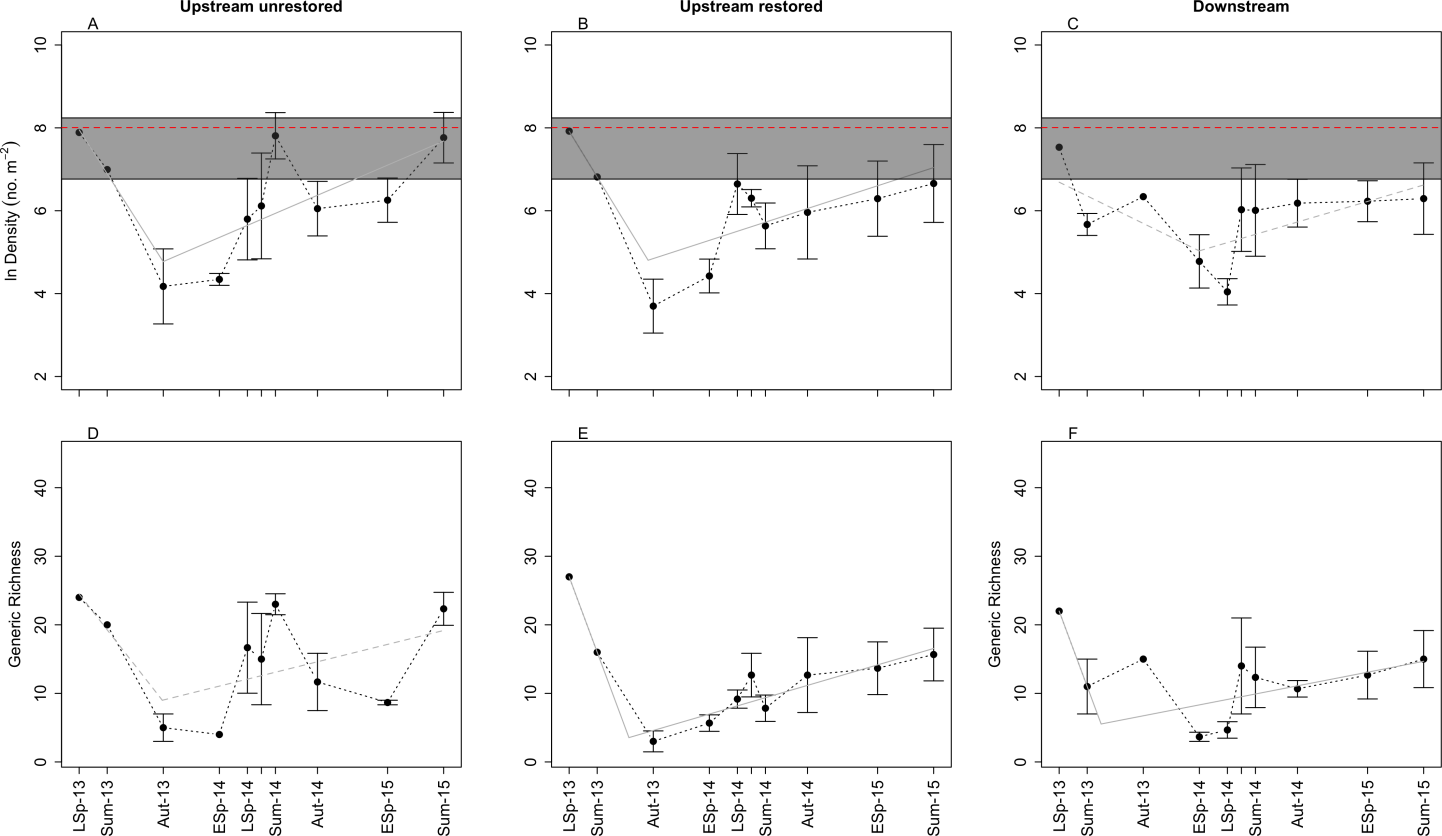
Plots of mean (±SE) benthic macroinvertebrate density (A–C) and generic richness (D–F) through time in the three study reaches: upstream restored (A, D), upstream unrestored (B, E), and downstream of former dam (C, F). Seasons represented are early spring (ESp), late spring (LSp), late autumn (Aut), and summer (Sum). In (A–C), the dashed horizontal red line and gray box surrounding this line denote the mean, miniumum, and maximum (bottom and top of the rectangles, respectively) macroinvertebrate density observed from June 2014–June 2015 in the upstream reference reach (*n* = 5) (Vent, 2015). Grey lines indicate breakpoint regression models for each reach: solid lines highlight the reaches with marginally signficant (*P* < 0.10) changes in slope (A, B, E, F), the dashed line indicates no significant change in slope (C, D; *P* > 0.10). Estimated breakpoints occurred ∼15 (A, B, E) to 19 (F) months after dam removal. Note: *y*-axis in (A–C) is ln-transformed, and July 2014 data are presented, but not labeled on the *x*-axes for visual clarity.

**Figure 3:**
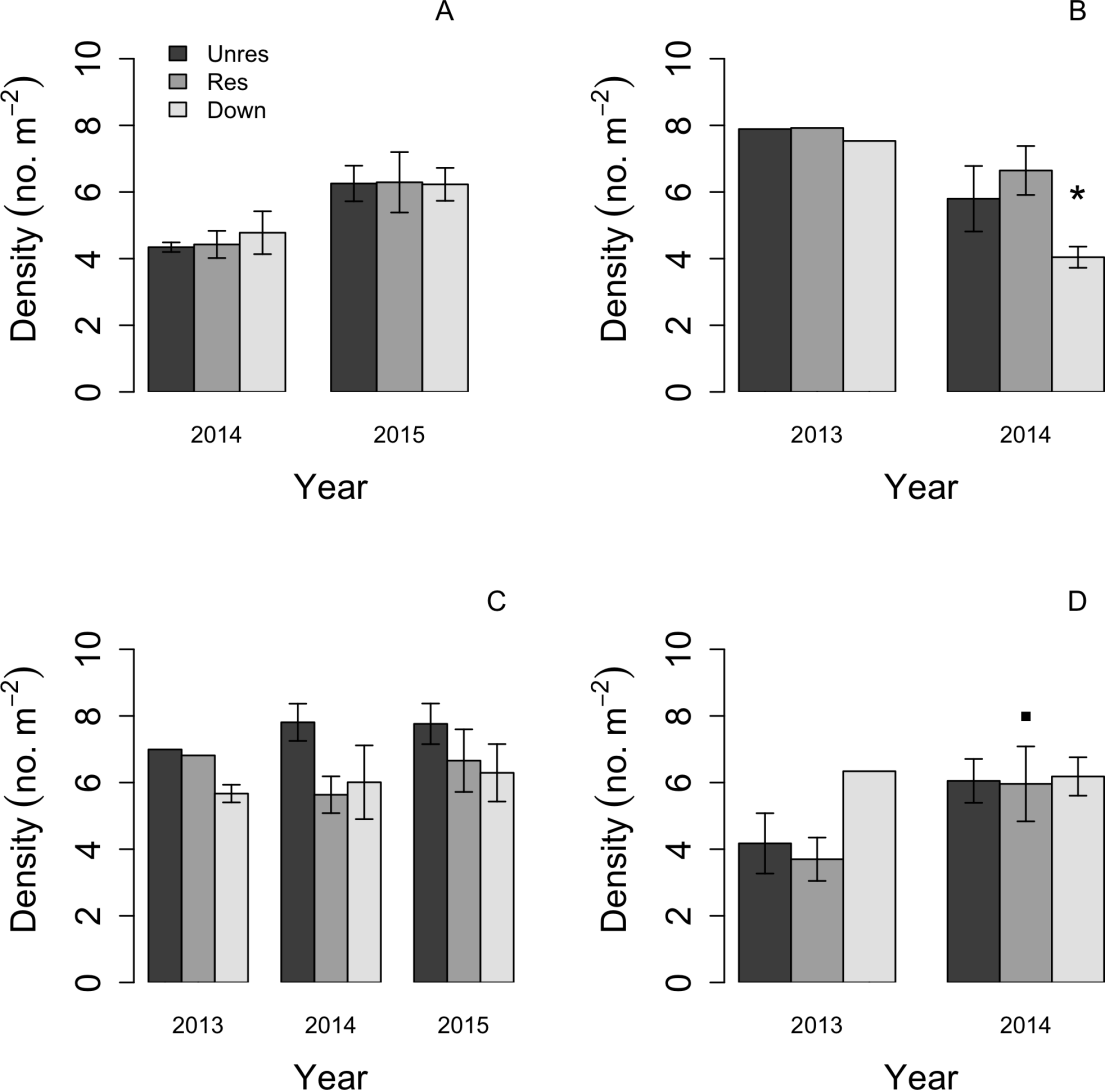
Barplots of mean (error bars: ±1 SE) ln-transformed macroinvertebrate density in each of the three reaches: upstream restored (gray bars), upstream unrestored (dark gray bars), and downstream (light gray bars) in the four seasonal periods across years (early spring [April, A], late spring [June, B], summer [August, C], and late autumn [November/December, D]). Based on orthogonal contrasts, macroinvertebrate densities significantly decreased in the downstream reach in late spring (Jun 2013–June 2014; *P* < 0.05, indicated by asterisk), but increased marginally in late autumn in the restored reach following dam removal (*P* = 0.089, indicated by filled square).

## Results

### Benthic macroinvertebrate density, diversity, and evenness

We identified 7,695 macroinvertebrates across all reaches and years. Mean (±SE) macroinvertebrate density was 4,359 ± 101 ind m^−2^ across all reaches and time points. There was a consistent pattern of decreasing macroinvertebrate density between 9 (summer 2013) and 15 (late autumn 2013) months after dam removal in all three reaches. We observed the lowest mean densities ∼19 (early spring 2014), ∼15 (late autumn 2013), and ∼21 (late spring 2014) months after dam removal in the upstream-unrestored, upstream-restored, and downstream reach, respectively. Macroinvertebrate densities subsequently increased through time at both upstream reaches beginning in early spring 2014 but not at the downstream reach (Figs. 2A–2C). The estimated changepoint (i.e., threshold response) occurred 15 months post-dam removal in both the upstream-unrestored (Davies test: *P* = 0.041) and restored (Davies test: *P* = 0.065) reaches. There was no significant change from decreasing to increasing macroinvertebrate density in the downstream reach (Davies test: *P* = 0.114). Although we had no true control data, macroinvertebrate densities generally rebounded to approach values found in a reference reach located directly below an intact dam (3,000 ± 635 ind m^−2^; *n* = 5; Vent, 2015) (Figs. 2A–2C) by the end of the study.

Macroinvertebrate density was highest in late spring during the first year of the study, but declined during this season in the second year in the downstream reach (orthogonal contrasts: *P* < 0.05; Fig. 3). Mean macroinvertebrate density was lowest in late autumn compared to the other seasons, but increased marginally in the following year in the upstream-restored reach (orthogonal contrasts: *P* = 0.089; Fig. 3). Summer densities also tended to increase, but not significantly, particularly in the upstream-unrestored reach (Fig. 3).

Similar to macroinvertebrate density, generic richness generally decreased between ∼9 (summer 2013) and ∼15 (late autumn 2013) months after dam removal, and richness subsequently increased through time (Figs. 2D–2F). In contrast to macroinvertebrate density, we detected no threshold response of generic richness in the upstream unrestored reach (Davies test: *P* = 0.141, Fig. 2D), while there was a significant changepoint in the richness versus time relationship 15 months following dam removal in the upstream-restored reach (Davies test: *P* = 7.365 × 10^−7^, Fig. 2E). The threshold for the change from increasing to decreasing generic richness was delayed (∼19 months after dam removal) in the downstream reach (Davies test: *P* = 0.056, Fig. 2F).

We found evidence for seasonal differences in generic richness, but not Shannon–Wiener diversity. Generic richness was lowest in late autumn during the first year of the study, but increased significantly during this season in the second year (orthogonal contrasts: *P* = 0.047; Figs. 2D–2F). Generic richness tended to be highest in late spring to summer, but was comparable across years (orthogonal contrasts; all *P* > 0.1; Figs. 2D–2F). Shannon-Wiener diversity (*H*′) also generally decreased between ∼9 and ∼15 months after dam removal, and subsequently increased through time (Figs. S2A–S2C). Similar to generic richness, we detected no threshold in the trajectory of *H*′ in the upstream unrestored reach (Davies test: *P* = 0.277, Fig. S2A), while there was a significant threshold response in *H*′ 15 months following dam removal in the upstream-restored reach (Davies test: *P* = 0.008, Fig. S2B). The change from decreasing to increasing *H*′ was delayed (∼19 months after dam removal) and significant in the downstream reach (Davies test: *P* = 0.017, Fig. S2C). In contrast to both density and generic richness, we found no evidence for seasonally distinct changes in Shannon–Wiener diversity (Figs. S2A–S2C; orthogonal contrasts: all *P* > 0.10). Generic evenness was relatively stable through time after dam removal, and this pattern was consistent among the three reaches (Fig. S3A–S3C). Generic evenness increased slightly in spring and summer in the upstream unrestored and downstream reaches (Figs. S3A–S3C).

### Macroinvertebrate community composition

**Figure 4:**
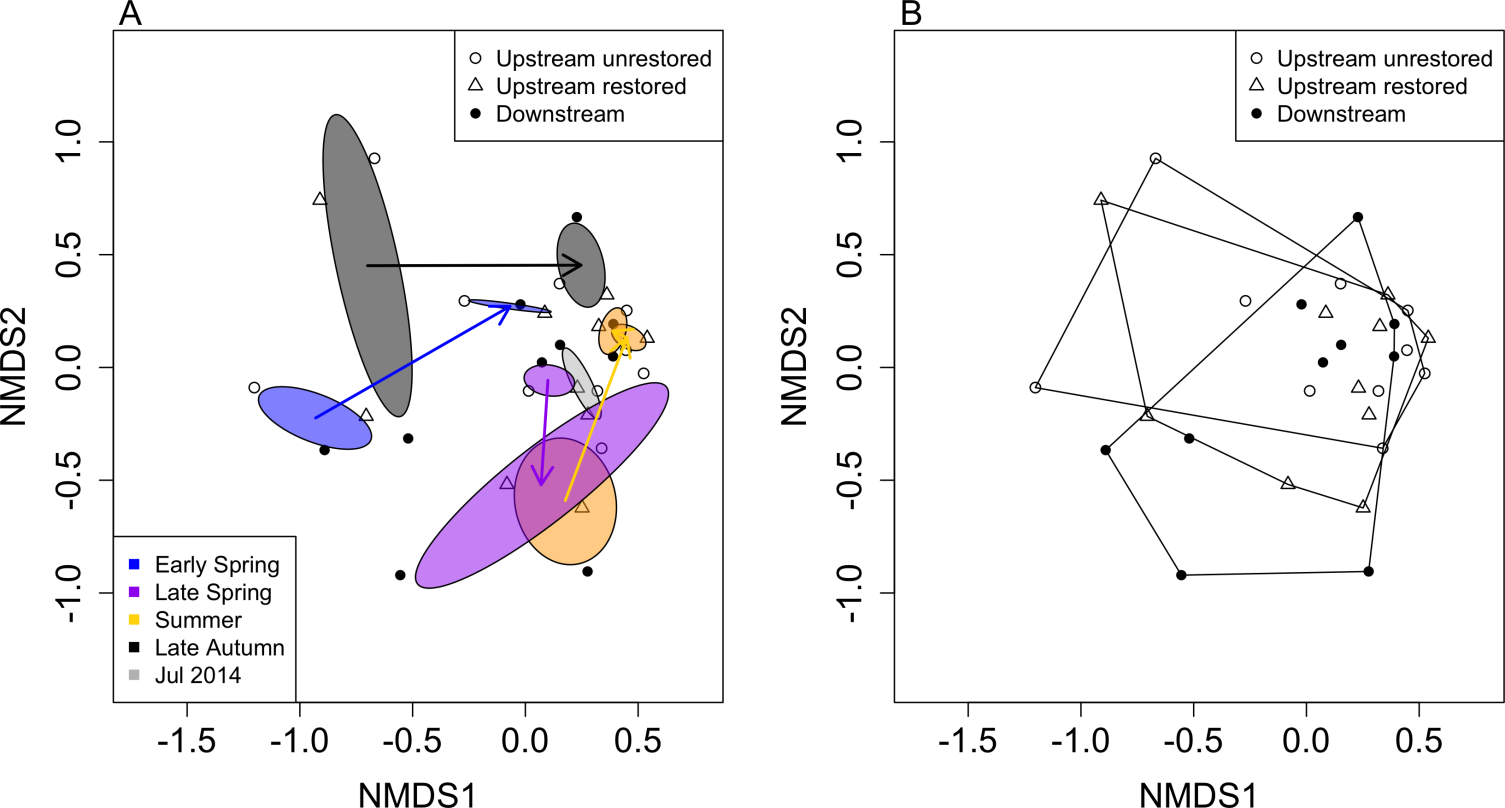
Non-metric multidimensional scaling (NMDS) ordination of reaches based on macroinvertebrate community data (by genus; stress = 0.186). Ellipses represent standard deviations around ordination points grouped by month after dam removal and are colour coded accordingly (A). Polygons in (B) denote the three reaches used in this study. Open circles, open triangles and closed circles indicate upstream-unrestored, upstream-restored, and downstream reaches, respectively.

The four most abundant families surveyed were Chironomidae (3,793 individuals), Hydropsychidae (1,736 individuals), Elmidae (625 individuals), and Baetidae (513 individuals), which represented 47, 23, 8, and, 7% of all macroinvertebrates, respectively. The taxonomic composition of the macroinvertebrate community differed across months after dam removal (NMDS: stress = 0.186; ANOSIM: *R* = 0.54, *P* = 0.001; Fig. 4A), but showed no differences among all reaches (ANOSIM: *R* = 0.0005, *P* = 0.452; Fig. 4B). Patterns of community structure were consistent between the chironomid assemblage and the complete macroinvertebrate community (see Fig. S4A and S4B). We found no evidence for changes in the relative abundance of the four most prevalent families through time (Figs. S5A–S5D; GLMs: all *P* > 0.1).

Macroinvertebrate community similarity responded differently depending on season and year (Fig. 4A). The largest distances between the same season but different years were for early spring (distance = 0.99), followed by late autumn (distance = 0.94), summer (distance = 0.77), and late spring (distance = 0.46). The smallest distance between years occurred between summers 2014 and 2015 (distance = 0.07). Within each season, macroinvertebrate community structure tended to become more similar to our final sampling date (summer 2015; Fig. 4A), where the distance between summer 2015 and late autumn 2014 was the smallest (0.32), followed by the distance between summer 2015 and early spring 2015 (0.47), then summer 2015 compared to late spring 2014 (0.75).

### Functional traits

We found no differences in the trajectory of relative functional-trait abundance among the three reaches; therefore we pooled the data across reaches for the functional analyses. In general, macroinvertebrate community functional traits shifted toward greater representation by traits such as semi- or univoltine life history, greater affinity for attaching to substrates, stronger armoring, and preferential use of erosional habitat (Table S1) based on declining relative abundance of multivoltine, free-ranging, poorly armored, depositional taxa (Figs. S6A–S6D; GLM: all *P* < 0.05) most strongly expressed by Chironomidae (Fig. S6E–S6H). The three most abundant FFGs were collector-gatherers (5,016 total individuals), collector-filterers (1,841 individuals), and herbivores (scrapers, piercers; 773 individuals), which represented 65, 24, and 10% of all macroinvertebrates, respectively. Among the functional feeding groups, relative abundance of collector-gatherers declined by ∼3.8% per month after dam removal (GLM: *P* = 2.16 × 10^−12^), while all others increased by a similar magnitude (i.e., collector-filterers, herbivores, predators, and shredders (detritivores); all *P* < 0.05; Table S1). There were also seasonal differences in the relative abundance of several functional traits (Table S2). Among our focal traits, there were significant declines in multivoltinism during early spring and late autumn (orthogonal contrasts: *P* = 0.0002, 0.019, respectively; Table S2). We also found decreased relative abundance of taxa that commonly occur in the drift, free-ranging taxa, poorly armored taxa, and those that prefer depositional habitat during all seasons except late spring (orthogonal contrasts: all *P* < 0.05; Table S2).

## Discussion

The nature of biological responses over time to perturbations like dam removal remains unresolved but is critical in defining the temporal scale of observations necessary to characterise ecological shifts and effectively manage river restorations. Our findings point to thresholds in macroinvertebrate response trajectories following dam removal. Further, we found that these thresholds were driven partly by seasonal patterns of macroinvertebrate density and richness, whereby both metrics recovered after dam removal, but the magnitude of the recovery varied seasonally. Finally, our findings also point to limited differences in macroinvertebrate communities between restored- and unrestored-upstream reaches, suggesting that engineered channel restoration may have limited short-term benefits for aquatic macroinvertebrates following lowhead dam removal.

Although we were unable to measure benthic macroinvertebrates immediately following dam removal, multiple responses—both taxonomic and functional—declined markedly between 9 and 15 months post-dam removal despite small differences in river discharge among these sampling periods (Figs. S1 and S7). Macroinvertebrate densities more closely tracked daily discharge estimates from the sampling day than antecedent flow conditions (mean 30-d discharge before sampling; Fig. S7). However, on the whole, densities were not strongly linked to discharge, emphasizing that other effects of dam removal also influence macroinvertebrate responses (Fig. S7). Beyond temporal differences in discharge, some of the variability in macroinvertebrate densities could be attributed to depressed benthic invertebrate densities during the winter months in temperate rivers (Murphy & Giller, 2000). Our findings suggest that macroinvertebrate responses can conflict within a short timespan, whereby the short-term effects of dam removal intensified seasonally low macroinvertebrate densities. Thus, the threshold responses we observed were likely driven by interactions among seasonal macroinvertebrate life histories, and both acute and gradual changes to the physical structure (e.g., channel depth, width; flow velocity) of the reaches impacted by dam removal (Figs. 1C–1D).

Macroinvertebrate diversity patterns were somewhat divergent between the upstream-unrestored reach and other two reaches (upstream-restored reach, downstream reach). For generic richness and *H*′, macroinvertebrate responses followed a similar trajectory as observed for density, with a decline starting at 9 months after dam removal and then an abrupt increase through the end of the study period. The patterns of macroinvertebrate richness were similar, but more muted and with lags in the response times in the downstream reach compared to the restored reach. However, as with density, these patterns were likely driven by seasonal changes through the course of the study. In some cases, comparisons across years within the same season suggest that macroinvertebrate responses to dam removal may be mediated by seasonal variability in streamflow (e.g., Fig. S1, but see above discussion) or water temperature. In our study system, temperature varied seasonally across all three reaches, with lowest temperatures in late autumn (−0.04 and 1.98 °C, in 2013 and 2014, respectively; *n* = 9 in both cases; Table S3) and highest temperatures in late spring of 2014 (25.8 °C; *n* = 9; Table S3). As with seasonal variability in streamflow and temperature, the magnitude of the differences in generic richness and *H*′ between summer collection periods and late autumn collection periods (Nov/Dec) in the upstream-restored reach, for instance, varied considerably.

The continuing rearrangement of the physicochemical habitat following dam removal has been proposed as a driver of macroinvertebrate responses (Doyle et al., 2005), and is likely a critical predictor in our study system as well. For example, flow rates increased by 2.88 × from June 2013 to June 2014 (see Fig. S1). However, the relationship between discharge and macroinvertebrate densities failed to fully explain the pattern, as some density decreases occurred with similar flows in subsequent sampling periods (Figs. S1 and S7). This pattern underscores the potential for broad-scale, season-specific effects of dam removal that affect macroinvertebrate community structure and density along with restored flow regimes. Additionally, Comes (2016) observed patterns of seasonal coarsening and fining of riverbed substrate: the upstream reaches exhibited overall coarsening and the downstream reach initially fined but coarsened for the overall study period. Although the joint effects of seasonal variability in streamflow and sediment regimes has not been commonly considered in the context of dam removal, our findings provide initial evidence that they may mediate macroinvertebrate response trajectories.

We found that seasonally distinct macroinvertebrate communities became more closely aligned with the summer communities we observed in our study, with the exception of late spring sampling periods. Summer macroinvertebrate communities were characterised by higher abundances of net-spinning caddisflies, baetid mayflies, and riffle beetles, suggesting that these taxa became relatively more abundant in both early spring and late autumn after dam removal. This result is consistent with the idea of taxonomic recovery to similar upstream-downstream communities that were formerly divergent as reported by Orr et al. (2008) and Tullos, Finn & Walter (2014). Although the taxonomic composition of the macroinvertebrate assemblage converged over time after dam removal, we observed no differences among the three study reaches, suggesting that the trajectory of macroinvertebrate community shifts were similar between post-dam management strategies (restored vs. unrestored). Stanley et al. (2002) observed that lotic taxa (e.g., net-spinning caddisflies, naidid worms, heptageniid mayflies) can dominate newly free-flowing reaches, and these communities differ from those in reaches remaining impounded, suggesting that once a river is returned to its free-flowing state, macroinvertebrate community structure may converge despite differing restoration strategies.

Similar to taxonomic differences through time, we observed concomitant changes in several functional traits during this study. These changes were evident either as a function of time after dam removal, or when comparing year-to-year differences in trait relative abundance within the same season. Our data suggest functional-trait changes could be more pronounced than taxonomic shifts as evidenced by stronger relationships between time after dam removal as compared to weak or no relationship for families, or orders. Notably, the functional traits that tended to decline in prevalence after dam removal included multivoltinism, free-ranging mobility (i.e., no attachment), poor armoring, depositional habitat use, and collector-gatherer feeding mode. In reference to potential taxonomic drivers of the functional patterns we observed, Chironomidae were the dominant family (>50% of individuals observed); many of the traits that tended to decrease the most are linked to this family. Thus, changes in their abundance could partly explain the differences in functional traits we observed (e.g., multivoltinism, depositional rheophily, poor armoring, collector-gatherer feeding mode, etc.) after dam removal.

## Conclusions

Overall, we observed variability in macroinvertebrate response trajectories by season, providing initial evidence that ecological responses to dam removal may be temporally variable and follow seasonally distinct recovery trajectories. These findings stress that assessments of newly free-flowing rivers should account for this type of temporal variability. For example, in dammed rivers, many natural disturbance events such as floods, dry downs, and overbank flows are eliminated or dampened, often for decades or more (Poff et al., 1997; Nilsson et al., 2005). Thus, as was the case in our study, fully evaluating pre-dam conditions are often unrealistic and quantifying “recovery” becomes challenging. Despite this challenge, our data show that signs of recovery can be detected, as evidenced by greater macroinvertebrate density during specific times of the year that approached values from a reference reach (i.e., late autumn, early spring). We suggest that, as conditions allow, managers and other practitioners might consider including off-season macroinvertebrate sampling as part of post-dam removal monitoring protocols, as periods of the year when macroinvertebrates are not at peak densities can provide important information on the degree of recovery.

Dam removal is increasingly considered a viable river-management approach, emphasizing the need for a robust understanding of its ecosystem-scale effects. In our system, we found no appreciable differences in macroinvertebrate responses between restored or unrestored reaches, although increased macroinvertebrate densities occurred more rapidly in the restored reach. This finding underscores highly variable macroinvertebrate responses to dam removal (Mbaka & Mwaniki, 2015), and suggests that their responses should be placed in the context of associated ecosystem-scale processes. Indeed, differences between restored vs. unrestored management approaches may still play out at higher levels in the food web (e.g., fish; Dorobek, Sullivan & Kautza, 2015) or over longer time scales (i.e., decades; Hansen & Hayes, 2012), stressing the importance of long-term monitoring and high-resolution food-web data following dam removal and associated restoration efforts. Overall, such information from the removal of lowhead dams will be an important step in developing comprehensive river management following the removal of in-stream infrastructure (Lorang & Aggett, 2005).

##  Supplemental Information

10.7717/peerj.3189/supp-1 Supplemental Information 1 Supporting Information

10.7717/peerj.3189/supp-2 Data S1 Raw Data

## Additional Information and Declarations

### Field Study Permissions

The following information was supplied relating to field study approvals (i.e., approving body and any reference numbers): Invertebrates were collected under Ohio Division of Wildlife Wild Animal Permit 15-49.

### Data Availability

The following information was supplied regarding data availability: The raw data has been supplied as a Supplementary File.
