# Supplementary material for: Seasonally distinct taxonomic and functional shifts in macroinvertebrate communities following dam removal
Source: PeerJ. 2017 Apr 6;5:e3189. doi: 10.7717/peerj.3189 (PMC5385129; doi:10.7717/peerj.3189)
Supplement: Supplemental Information 1 [file peerj-05-3189-s001.docx]

**Supporting Information: Appendix S1**

Sensitivity Analysis: Analyzing Data With and Without Chironomidae

We found that the macroinvertebrate community structure without Chironomidae differed significantly between successive months following dam removal (stress = 0.187, ANOSIM: *R* = 0.508, *P* = 0.001; Fig. S4A), but were similar among the unrestored, restored, and downstream reaches (OR2, OR3 and OR5, respectively; ANOSIM: *R* = -0.029, *P* = 0.667; Fig. S4B).

We found that several of the changes in functional traits we observed for the entire community were undetectable when we analyzed the functional traits of the non-chironomid taxa alone. Specifically, we observed no changes in the relative abundance of multivoltinism, depositional rheophily, or burrowing habit (Fig. S6E-G). In contrast, collector-gatherers still declined even in the absence of chironomids (Fig. S6H). Similarly, patterns of prevalence of traits linked to the ability to attach to substrates, and declines in poorly armored taxa persisted when chironomids were excluded from the functional trait analysis (Table S1).

**Fig. S1.** Mean daily discharge (m^3^ s^-1^) recorded on the Olentangy River near Worthington, Ohio (U.S. Geological Survey station number 03226800). Arrows indicate timing of dam removal, or sampling dates for the study. Median discharge from dam removal until the final sampling point was 5.9 m^3^ s^-1^ and ranged from 0.5 to 152.5 m^3^ s^-1^ (U.S. Geological Survey). Median discharge was ~2× higher in the first full year after dam removal (2013) and the final year of the study (2015) compared to the middle year (2014).

**
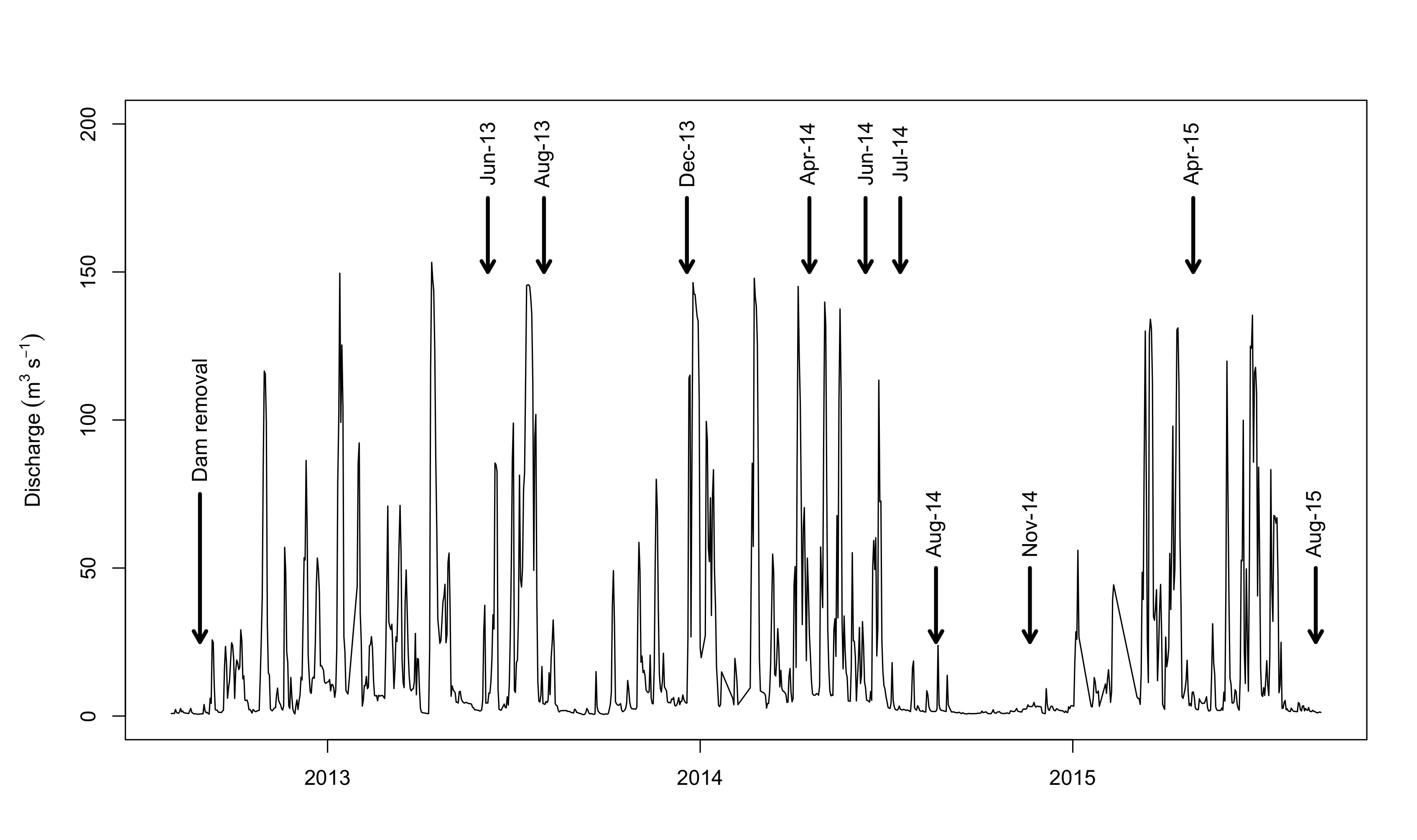
**

**Fig. S2A-C.** Mean (±SE) Shannon-Weiner diversity (*H*’) for the three reaches sampled in this study: upstream unrestored (A), upstream restored (B), and downstream of former dam (C). Seasons represented are early spring (ESp), late spring (LSp), late autumn (Aut), and summer (Sum). July 2014 data are plotted but not labeled on the axes for visual clarity. Grey lines indicate breakpoint regression models for each reach: solid lines highlight the reaches with marginally signficant (*P* < 0.10) changes in slope, the dashed line indicates no significant change in slope (*P* > 0.10).

**
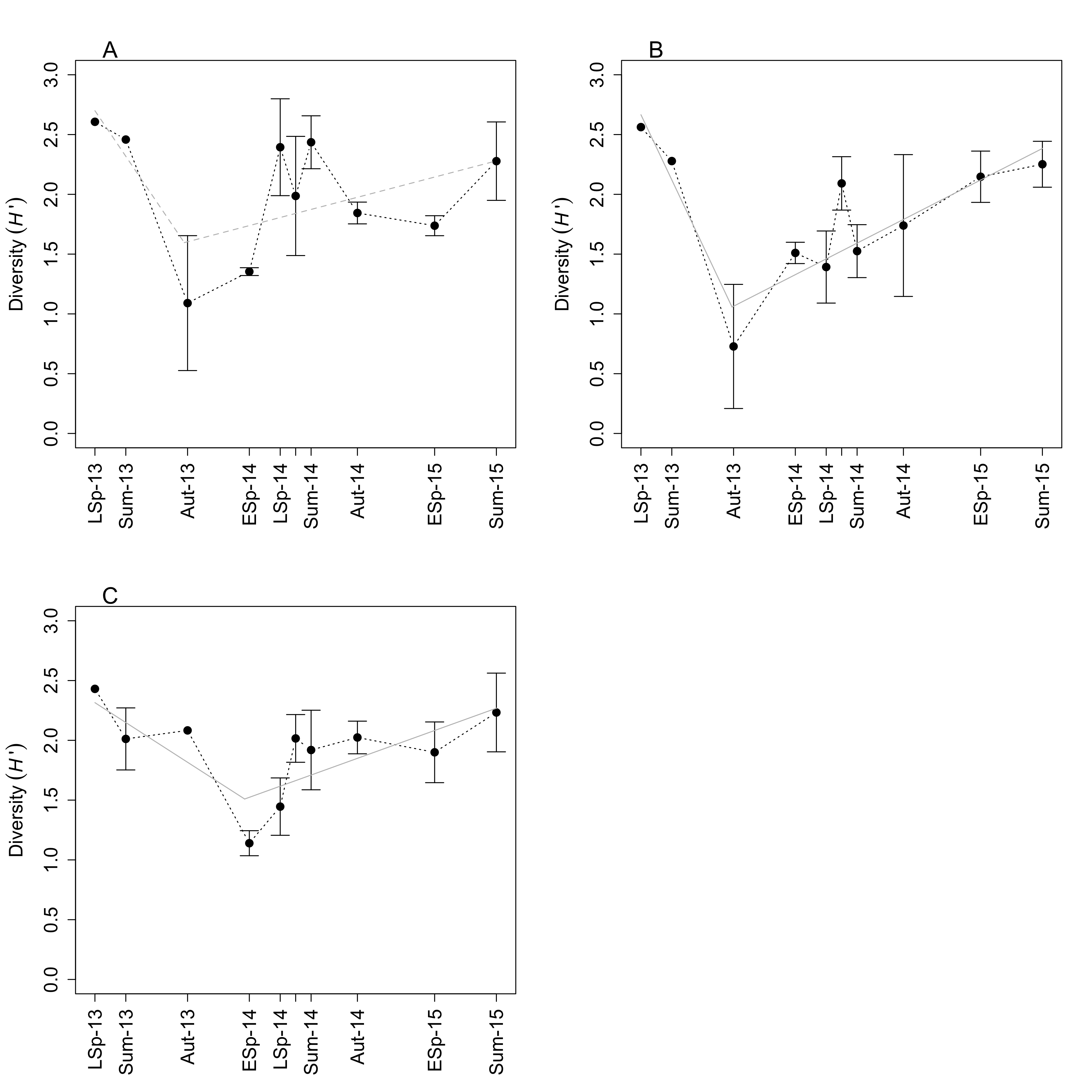
**

**Fig. S3A-C.** Mean (±SE) generic evenness for the ten time intervals following dam removal in the three study reaches (upstream unrestored [A], upstream restored [B], and downstream of former dam [C]). Seasons represented are early spring (ESp), late spring (LSp), late autumn (Aut), and summer (Sum). July 2014 data are plotted but not labeled on the axes for visual clarity. General linear models indicated no significant increases or decreases in generic evenness as a function of time since dam removal (all *P* > 0.1; data not shown).

**
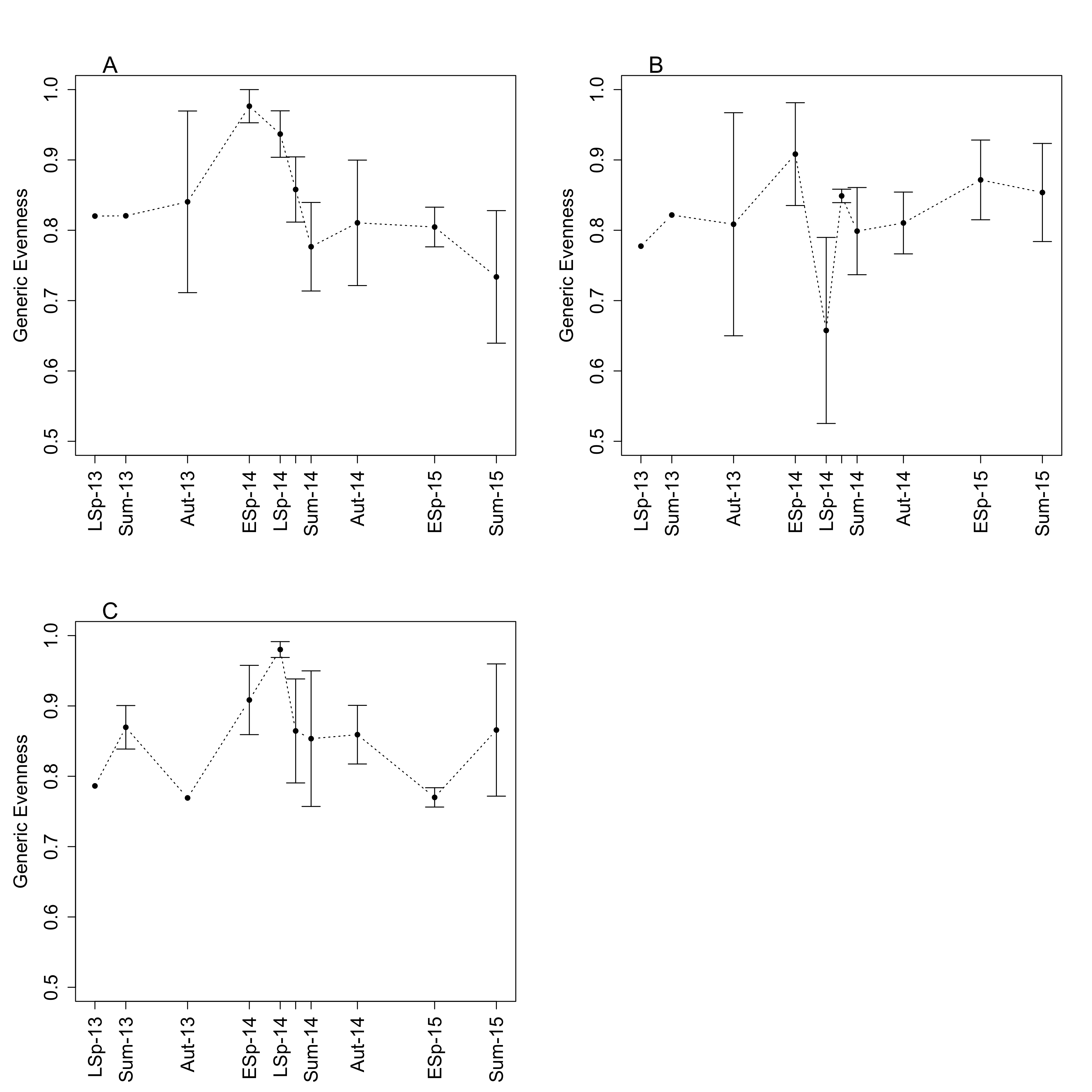
**

**Fig. S4A,B.** Ordination plots for macroinvertebrate community excluding Chironomidae (stress = 0.187). Ellipses or polygons denote groupings of distances by month (A) and by reach (B). Colours correspond to the month of sampling (e.g., black denotes autumn 2013, and autumn 2014, respectively). Open circles, open triangles and closed circles indicate the upstream reaches (passive = unrestored, active = restored) and the downstream reach, respectively.

**
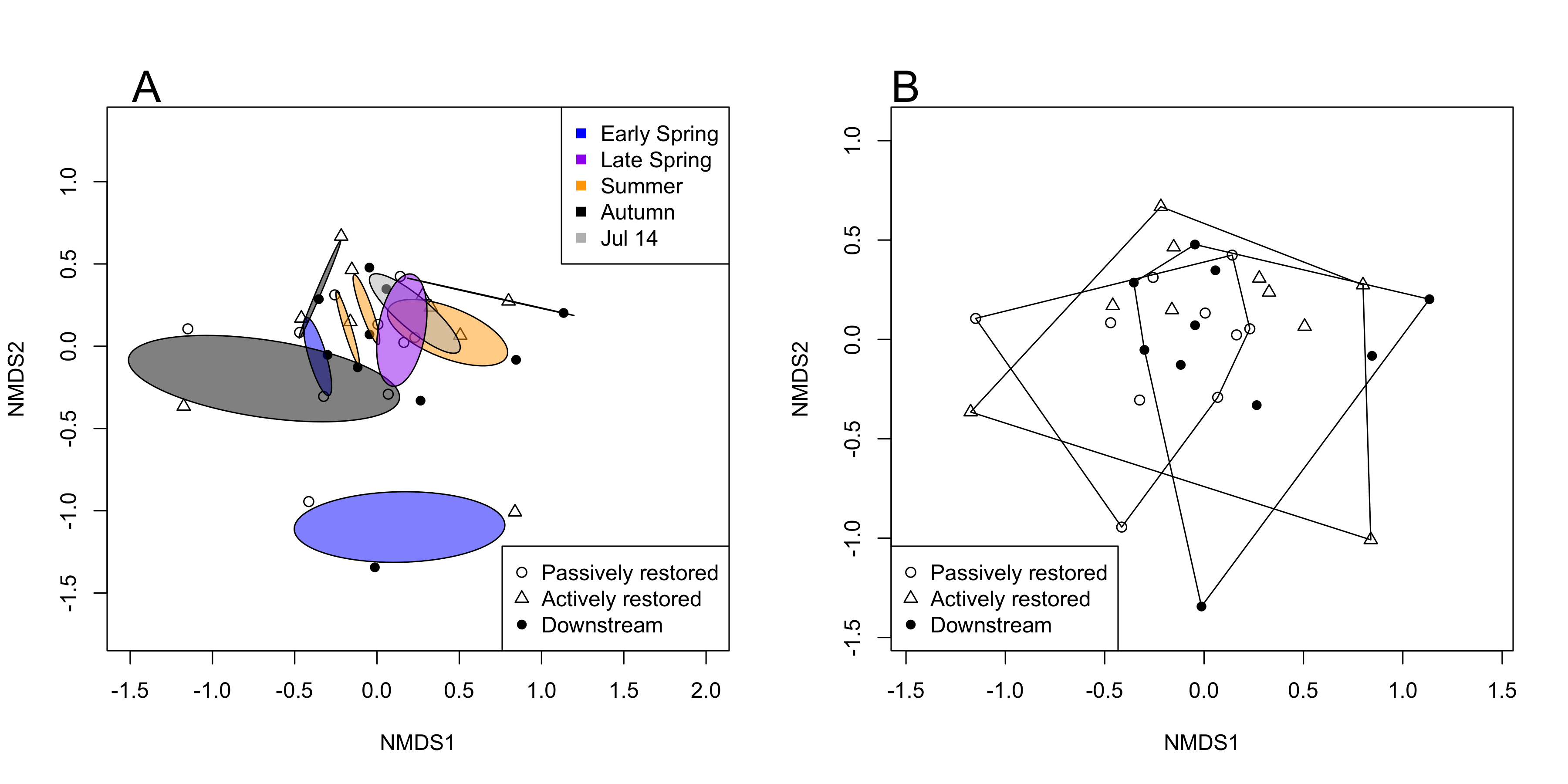
**

**Fig. S5A-D.** Mean (±SE) relative abundance of the four most abundant families observed during this study (Chironomidae [A], Hydropsychidae [B], Baetidae [C], and Elmidae [D], respectively) as a function of time after dam removal (months). Seasons represented are early spring (ESp), late spring (LSp), late autumn (Aut), and summer (Sum). Hydropsychidae (B) and Baetidae (C) were periodically not observed, therefore no data is plotted for these seasons (e.g., Baetidae, late autumn 2013). July 2014 data are plotted but not labeled on the axes for visual clarity. Note: there were no differences in these patterns among reaches, so the analysis reflects pooled relative abundance across all three reaches (*P* > 0.10 in all panels), and *y*-axis is presented on an ln-transformed scale.

**
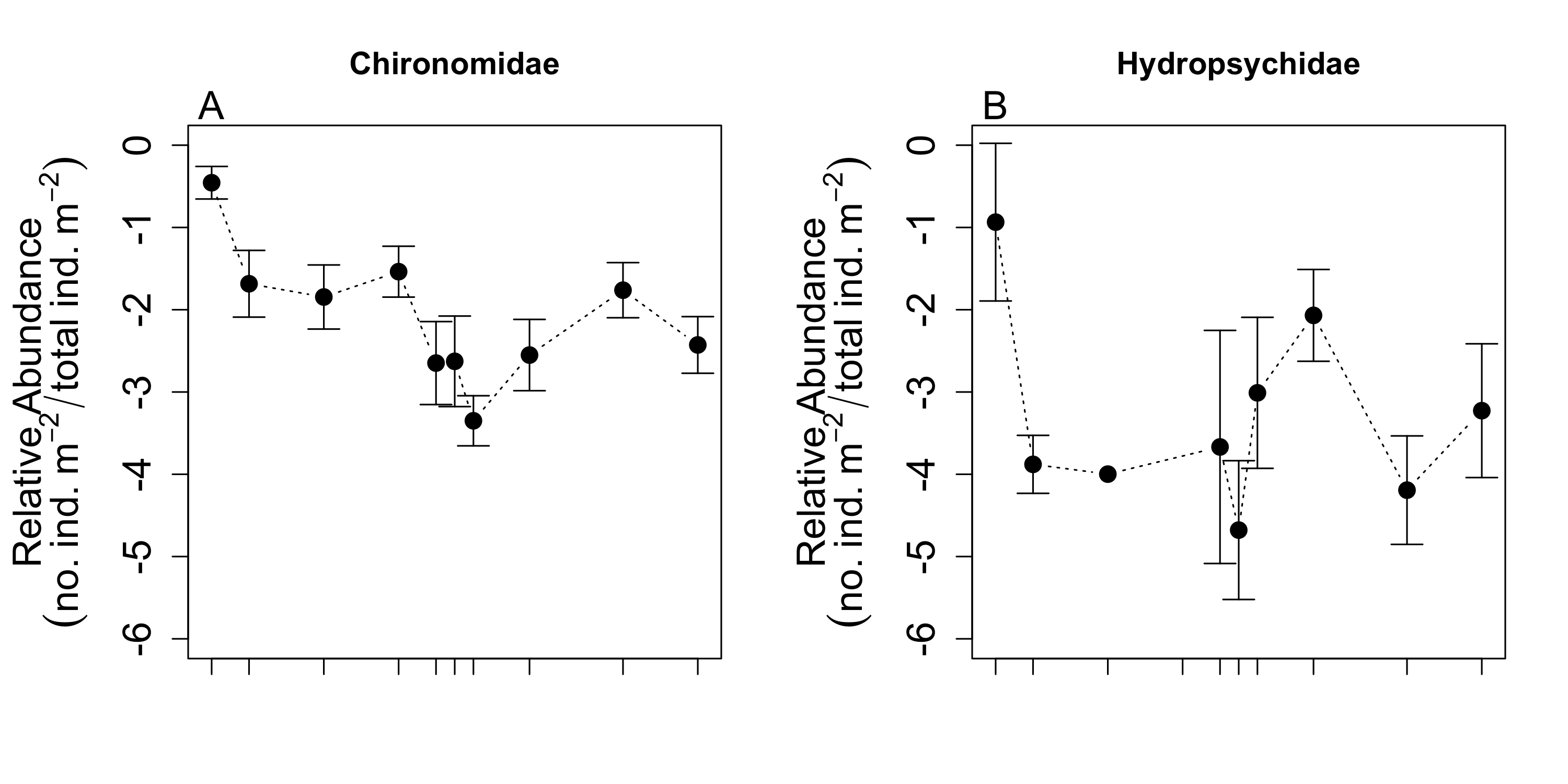
**

**
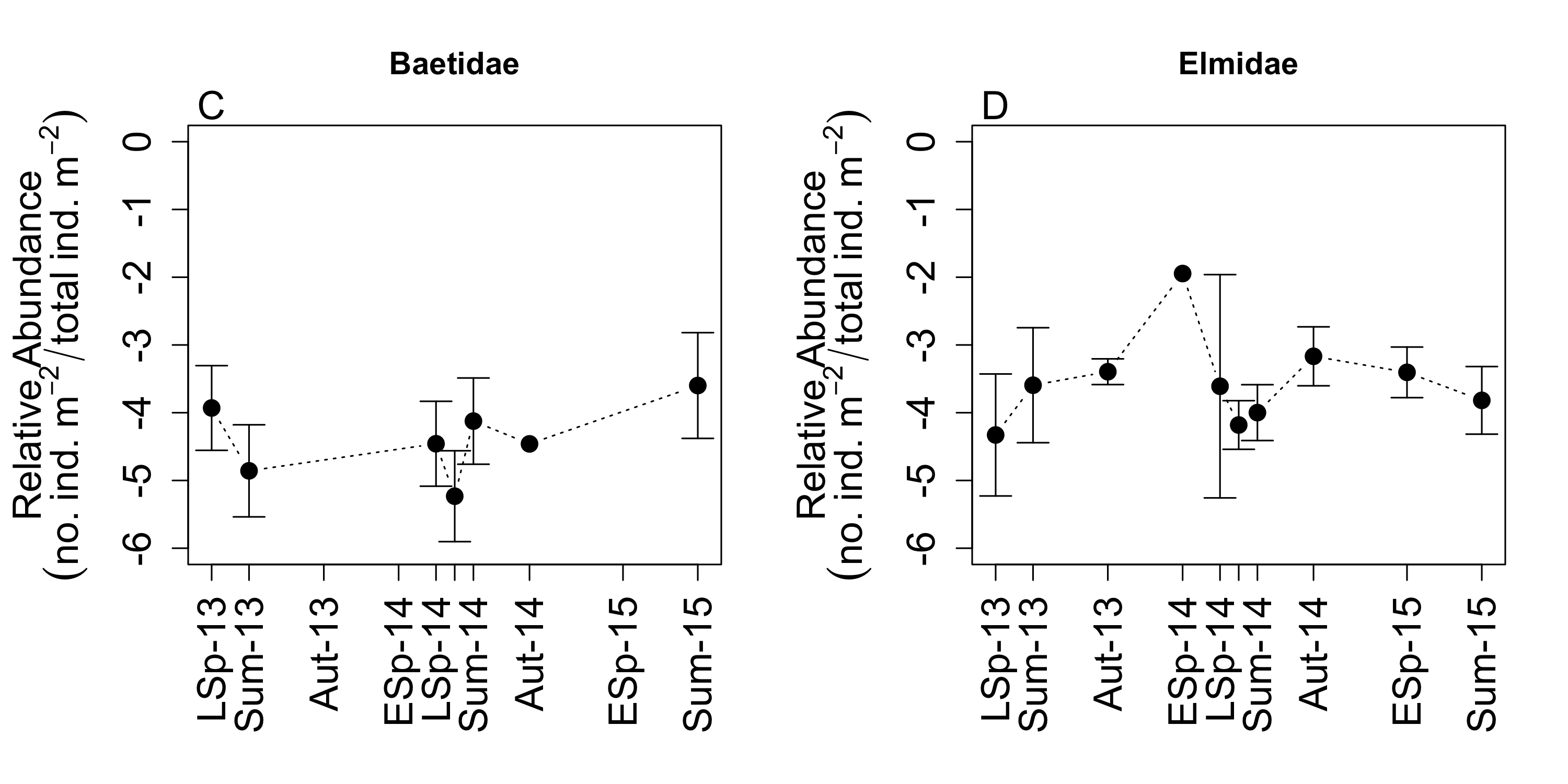
**

**Fig. S6A-H.** Mean (±SE) relative abundance of four traits characterized by significant declines in the post-dam period when analyzed for: (1) the complete macroinvertebrate assemblage, where we found significant declines (general linear models: all *P* < 0.05) in multivoltinism (A), depositional rheophily (B), burrowing habit (C), and collector-gatherer feeding mode (D), and (2) when individuals in the family Chironomidae were removed (E-H, *P* > 0.10). July 2014 data are plotted but not labeled on the axes for visual clarity. Note: there were no differences in these patterns among reaches, so the analysis reflects pooled relative abundance across all three reaches.

**
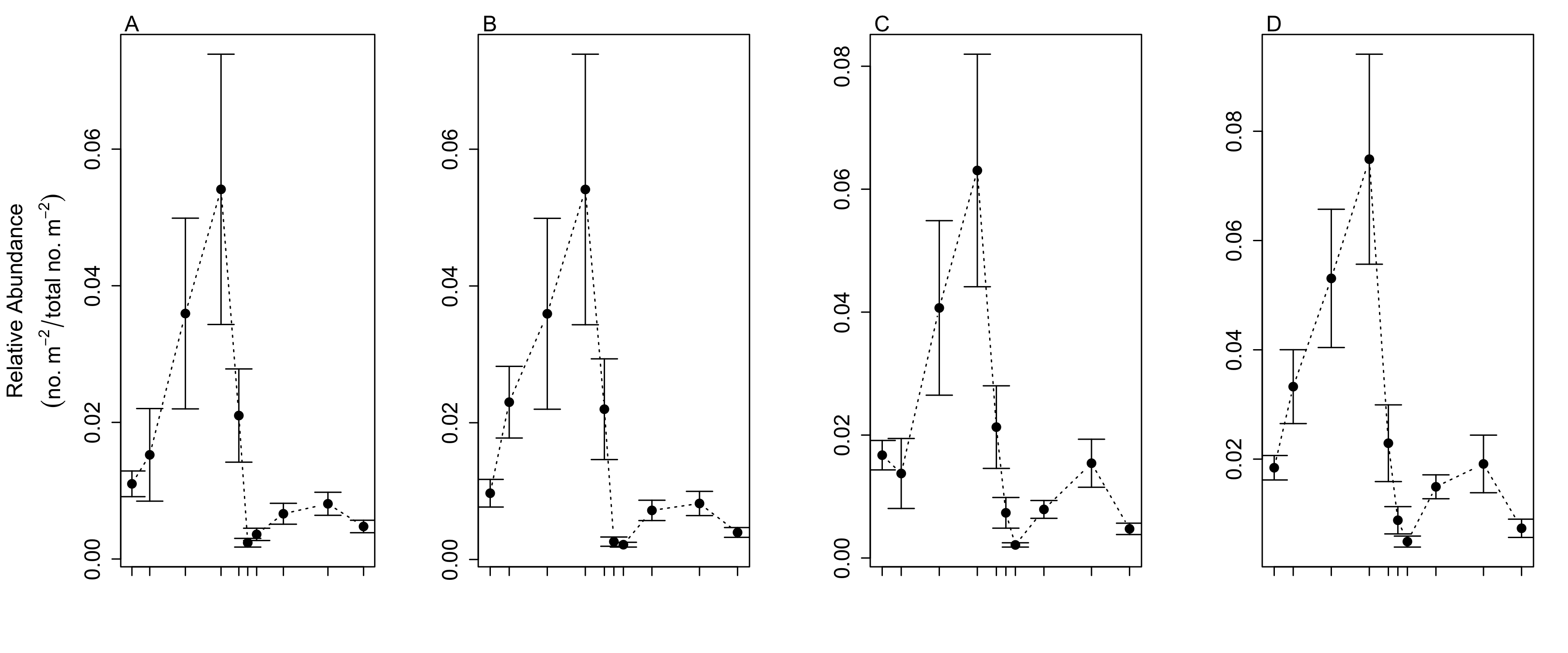
**


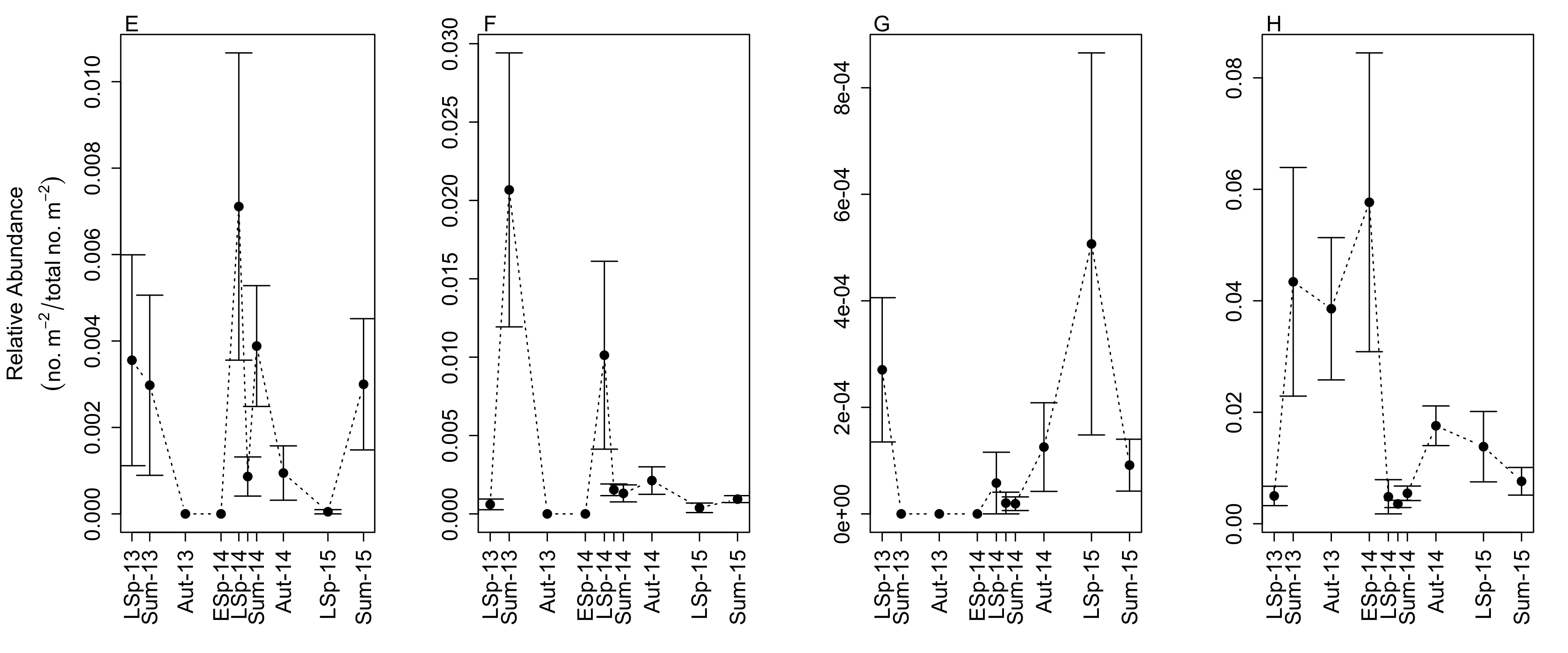


**
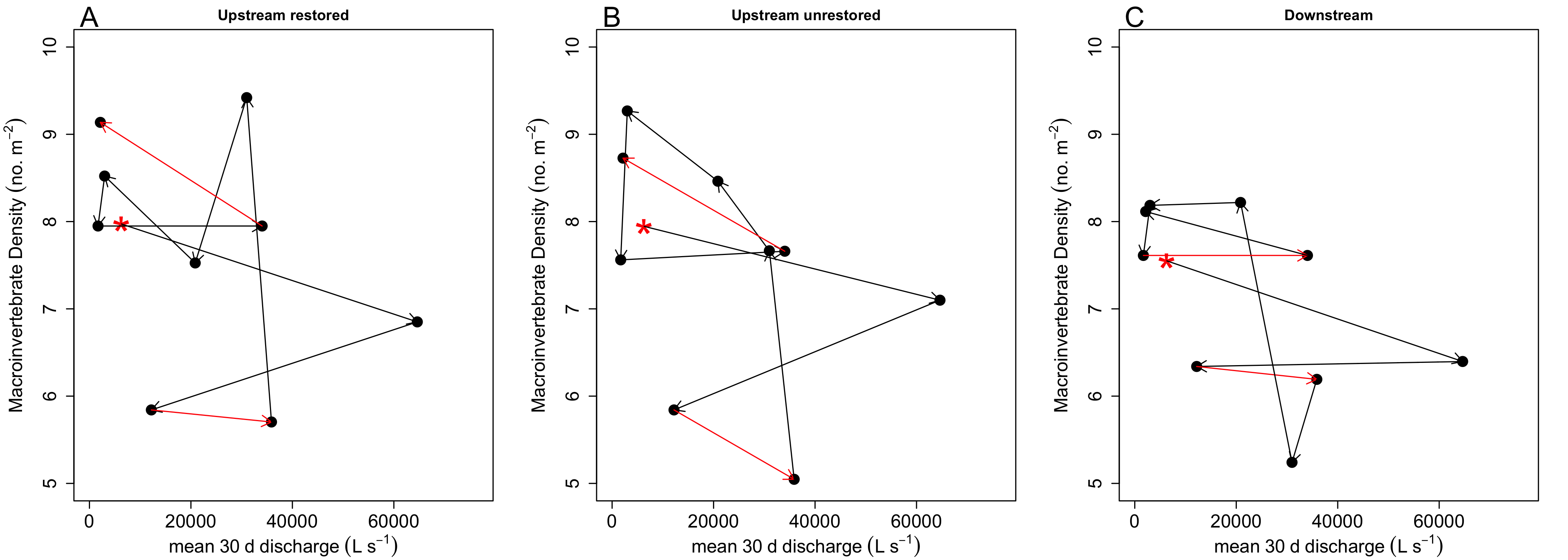
 Figure S7A-C.** Ln-transformed mean macroinvertebrate density plotted against mean discharge for the 30-d period before each sampling date in the upstream restored (A), upstream unrestored (B), and downstream reaches (C) used in this study. Macroinvertebrate density was generally unrelated to 30-d discharge (simple linear regression; *R*^2^ = 0.02; *P* = 0.72). We have emphasized that many decreases observed could be attributable to seasonal declines by showing the hysteresis of the macroinvertebrate density and how this changed throughout our study. Red arrows highlight winter-to-early spring minima observed across substantially different river discharge. Black arrows connect the preceding macroinvertebrate density value to the next data point. The starting point of the study is indicated with a red asterisk.

**Table S1.** Analysis of relative abundance of eight trait categories based on Poff et al. (2006). Parameter estimates and associated *P*-values of general linear models with and without invertebrates of the family Chironomidae, relating the relative abundance of trait categories and time after dam removal (months). N/A denotes traits that were not observed within the macroinvertebrate community throughout the study.

|  |  | With Chironomidae | | Without Chironomidae | |
| --- | --- | --- | --- | --- | --- |
| Trait | Trait category | Estimate | P-value | Estimate | P-value |
| Voltinism | semi- | -0.0036 | 0.657 | -0.0011 | 0.903 |
|  | uni- | -0.0152 | 0.183 | -0.0238 | 0.071 |
|  | bi-/multi- | **-0.0274** | **0.017** | -0.0013 | 0.918 |
|  |  |  |  |  |  |
| Occurrence in drift | Rare | -0.0077 | 0.298 | -0.0124 | 0.136 |
|  | Common | -0.0053 | 0.615 | -0.0094 | 0.422 |
|  | Abundant | **-0.0271** | **0.010** | -0.0046 | 0.699 |
|  |  |  |  |  |  |
| Attachment | None (free-ranging) | **-0.0428** | **9.62 * 10^-8^** | **-0.0312** | **0.001** |
|  | Some (sessile, sedentary) | **0.0416** | **1.74 * 10^-4^** | **0.0284** | **0.034** |
|  | Both | N/A | N/A | N/A | N/A |
|  |  |  |  |  |  |
| Armoring | None (soft-bodied forms) | **-0.0385** | **4.97 * 10^-8^** | **-0.0243** | **0.009** |
|  | Poor (heavily sclerotized) | **0.0341** | **4.90 * 10^-4^** | 0.0218 | 0.099 |
|  | Good (e.g., cased caddisflies) | **0.0363** | **2.10 * 10^-4^** | 0.0206 | 0.119 |
|  |  |  |  |  |  |
| Rheophily | Depositional only | **-0.0357** | **9.19 * 10^-6^** | -0.0140 | 0.113 |
|  | Depositional and erosional | 0.0165 | 0.14051 | -0.0094 | 0.453 |
|  | Erosional | **0.0325** | **0.004** | 0.0086 | 0.492 |
|  |  |  |  |  |  |
| Thermal preference | Cold stenothermal or cool eurythermal | -0.0103 | 0.121 | -0.0093 | 0.187 |
|  | Cool/warm eurythermal | **-0.0287** | **0.002** | -0.0183 | 0.066 |
|  | Warm eurythermal | 0.0093 | 0.320 | 0.0071 | 0.473 |
|  |  |  |  |  |  |
| Habit | Burrow | **-0.0314** | **3.04 * 10^-7^** | 0.0008 | 0.897 |
|  | Climb | **0.0310** | **3.13 * 10^-4^** | -0.0017 | 0.843 |
|  | Sprawl | **0.0218** | **0.011** | -0.0140 | 0.103 |
|  | Swim | 0.0140 | 0.102 | **-0.0291** | **0.001** |
|  | Skate | **0.0328** | **1.41 * 10^-4^** | 0.0017 | 0.845 |
|  |  |  |  |  |  |
| Trophic habit | Collector-gatherer | **-0.0388** | **2.16 * 10^-12^** | **-0.0200** | **0.004** |
|  | Collector-filterer | **0.0411** | **9.25 * 10^-8^** | **0.0234** | **0.018** |
|  | Herbivore (scraper, piercer, and shredder) | **0.0277** | **2.75 * 10^-4^** | -0.0005 | 0.963 |
|  | Predator | **0.0343** | **7.66 * 10^-6^** | 0.0180 | 0.068 |
|  | Shredder (detritivores) | **0.0378** | **8.34 * 10^-7^** | 0.1857 | 0.060 |

**Table S2.** Seasonal comparisons of specific trait modalities of macroinvertebrates after dam removal. Significant changes in the relative abundance of traits based on orthogonal contrasts by season (e.g., June 2013 vs. June 2014) are emphasized with bold text. The direction of the change is indicated by an up or down arrow (for an increase or decrease in relative abundance, respectively). N/A indicates where no macroinvertebrates with that specific trait were observed, and n.d. indicates where there was no difference between years within the same season for a trait.

| Trait | Trait category | Seasonal Comparisons | Direction of change | |
| --- | --- | --- | --- | --- |
| Voltinism | semi- | n.d. | n.d. |  |
|  | uni- | late spr. | 🡻 |  |
|  | **bi-/multi-** | **early spr.; late aut.** | 🡻 |  |
|  |  |  |  |  |
| Occurrence in drift | Rare | n.d. |  |  |
|  | Common | late spr. | 🡻 |  |
|  | **Abundant** | **early spr.; late aut.; late sum.** | 🡻 |  |
|  |  |  |  |  |
| Attachment | **None (free-ranging)** | **early spr.;late aut.; late sum.** | 🡻 |  |
|  | Some (sessile, sedentary) | late spr. | 🡻 |  |
|  | Both | N/A | N/A |  |
|  |  |  |  |  |
| Armoring | **None (soft-bodied forms)** | **early spr.;late aut.; late sum.** | 🡻 |  |
|  | Poor (heavily sclerotized) | n.d. | n.d. |  |
|  | Good (e.g., some cased caddisflies) | n.d. | n.d. |  |
|  |  |  |  |  |
| Rheophily | **Depositional only** | **early spr.; late aut.; late sum.** | 🡻 |  |
|  | Depositional and erosional | early spr.; late spr. | 🡻 |  |
|  | Erosional | n.d. | n.d. |  |
|  |  |  |  |  |
| Thermal preference | Cold stenothermal or cool eurythermal | n.d. | n.d. |  |
|  | **Cool/warm eurythermal** | **early spr.;late aut.; late sum.** | 🡻 |  |
|  | **Warm eurythermal** | **late spr.** | 🡻 |  |
|  |  |  |  |  |
| Habit | Burrow | early spr.;late aut.; late sum. | 🡻 |  |
|  | Climb | late spr. | 🡹 |  |
|  | **Sprawl** | **late sum.** | 🡻 |  |
|  | **Cling** | **late spr.; late sum.** | 🡻 |  |
|  | **Swim** | **late sum.** | 🡹 |  |
|  |  |  |  |  |
| Trophic habit | **Collector-gatherer** | **early spr.;late aut.; late sum.** | 🡻 |  |
|  | Collector-filterer | late spr. | 🡻 |  |
|  | **Herbivore (scraper, piercer, and shredder)** | **late sum.** | 🡻 |  |
|  | Predator | n.d. | n.d. |  |
|  | **Shredder (detritivores)** | **late aut.** | 🡻 |  |

**Table S3.** Mean temperatures (*n* = 3) from each reach (upstream unrestored, upstream restored, downstream) for each sampling period. Temperature measurements were not taken during April or July 2014 (indicated by n.a. = not available). Note that seasonal means pooled by reach are reported in the text.

| Reach | Month | Year | Season | Temperature (°C) |
| --- | --- | --- | --- | --- |
| Upstream unrestored | June | 2013 | Late Spring | 24.24 |
| Upstream unrestored | August | 2013 | Summer | 22.79 |
| Upstream unrestored | December | 2013 | Late Autumn | -0.28 |
| Upstream unrestored | April | 2014 | Early Spring | 10.71 |
| Upstream unrestored | June | 2014 | Late Spring | 24.30 |
| Upstream unrestored | July | 2014 | Summer | n.a. |
| Upstream unrestored | August | 2014 | Summer | 24.61 |
| Upstream unrestored | November | 2014 | Late Autumn | 1.71 |
| Upstream unrestored | April | 2015 | Early Spring | 11.79 |
| Upstream unrestored | August | 2015 | Summer | 20.58 |
| Upstream restored | June | 2013 | Late Spring | 22.77 |
| Upstream restored | August | 2013 | Summer | 22.42 |
| Upstream restored | December | 2013 | Late Autumn | 0.06 |
| Upstream restored | April | 2014 | Early Spring | 10.54 |
| Upstream restored | June | 2014 | Late Spring | 27.51 |
| Upstream restored | July | 2014 | Summer | n.a. |
| Upstream restored | August | 2014 | Summer | 25.57 |
| Upstream restored | November | 2014 | Late Autumn | 2.07 |
| Upstream restored | April | 2015 | Early Spring | 12.78 |
| Upstream restored | August | 2015 | Summer | 21.75 |
| Downstream | June | 2013 | Late Spring | 21.95 |
| Downstream | August | 2013 | Summer | 19.73 |
| Downstream | December | 2013 | Late Autumn | 0.11 |
| Downstream | April | 2014 | Early Spring | n.a. |
| Downstream | June | 2014 | Late Spring | 24.33 |
| Downstream | July | 2014 | Summer | n.a. |
| Downstream | August | 2014 | Summer | 25.05 |
| Downstream | November | 2014 | Late Autumn | 2.15 |
| Downstream | April | 2015 | Early Spring | 14.13 |
| Downstream | August | 2015 | Summer | 22.76 |
